# Predictors of Pap smear testing uptake among women in Lagos, Nigeria

**DOI:** 10.3332/ecancer.2022.1368

**Published:** 2022-03-31

**Authors:** Kehinde S Okunade, Sarah John-Olabode, Adebola A Adejimi, Yusuf A Oshodi, Benedetto Osunwusi, Aloy O Ugwu, Ayodeji Adefemi, Rose I Anorlu

**Affiliations:** 1 Department of Obstetrics & Gynaecology, College of Medicine, University of Lagos, Surulere, Lagos, Nigeria; 2 Department of Obstetrics & Gynaecology, Lagos University Teaching Hospital, Surulere, Lagos, Nigeria; 3 Department of Haematology and Blood Transfusion, College of Medicine, University of Lagos, Surulere, Lagos, Nigeria; 4 Department of Community Health and Primary Care, College of Medicine, University of Lagos, Surulere, Lagos, Nigeria; 5 Department of Obstetrics & Gynaecology, Lagos State University College of Medicine, Ikeja, Lagos, Nigeria; 6 Department of Obstetrics & Gynaecology, Lagos State University Teaching Hospital, Ikeja, Lagos, Nigeria

**Keywords:** cervical cancer, health promotion, mHealth-Cervix, Nigeria, Pap smear uptake

## Abstract

We assessed the predictors of Pap smear testing uptake within 6 months after cervical cancer prevention education among women in Lagos, Nigeria. This was a prospective follow-up study conducted as part of the ‘mHealth-Cervix trial’ in the two teaching hospitals in Lagos, Nigeria, between August 2020 and April 2021. Participants were followed up for 6 months after pre-enrolment cervical cancer prevention education. The potential socio-demographic and clinical predictors of Pap smear testing uptake during the 6-month follow-up were tested using the predictive model in a binary logistic regression analysis. Statistical significance was reported as *p* < 0.05. The rate of Pap smear testing uptake during the 6-month follow-up was 35.7%. Following the adjustments in the final multivariate analysis, participants’ previous awareness of Pap smearing (RR = 6.92, 95% CI: 8.37–56.68, *p* = 0.001) and attendance at the general outpatient clinic during the period of follow-up (RR = 11.22, 95% CI: 1.54–81.51, *p* = 0.017) independently predict Pap smear testing uptake. We will, therefore, explore the impact of continuous provision of health promotion on cervical cancer prevention and its effect in the context of routine clinical care in our next implementation research agenda. We recommend, in the meantime, that regular health education of women on cervical cancer prevention by healthcare providers should be further reinforced as an integral part of health promotion in clinics to reduce the burden of cervical cancer in most low- and middle-income settings.

## Introduction

Cervical cancer is the most common cancer of the reproductive tract and a leading cause of cancer deaths among women in sub-Saharan African countries, including Nigeria [1]. An estimated 570,000 new cases of cervical cancer and over 300,000 related deaths were reported in 2018, with 70% of these deaths occurring in developing countries [2].

The incidence of the disease and its associated mortality have remained high in many resource-limited settings, due to the lack of organised regular screening programmes to detect and treat cervical precursor lesions [3].

Screening with Papanicolaou (Pap) smear has remained an effective public health intervention for reduction of the incidence and mortality of cervical cancer disease [4,5]. However, this is largely applied through opportunistic screening in most developing countries, including Nigeria, as payment for testing is usually done out of pocket [6–8]. This method is less effective as it targets only women who come in contact with healthcare providers for other reasons in the healthcare facility [6,9]. Furthermore, in areas with limited access to screening tests, various studies have also reported very low levels of Pap smear [5,8,10,11]. Approximately 50%–90% of the women who developed cervical cancer or died as a result of the disease have never been screened [6], and even more worrisome is that a large proportion of these women were never aware of cervical cancer or its available prevention strategies [5,12,13].

Several factors are reported to influence the uptake of Pap smear testing and these include a high level of education, reduced perception of risk of developing cervical cancer, lack of knowledge about the benefits of Pap smear and lack of familiarity with screening locations [7,14–16]. The healthcare providers also play a significant role in improving the knowledge of cervical cancer prevention and uptake of any of its screening methods [17]. This is because the previous lack of awareness due to the non-recommendation of Pap smear testing by healthcare practitioners is a major reason why women are unable to take up screening [17]. Therefore, this study will seek to improve cervical cancer screening awareness by providing pre-enrolment cervical cancer prevention education that highlights the benefits of Pap test screening in women. These women were followed up individually for 6 months to assess their willingness to return for Pap smear testing. The outcome of this study could be used to suggest effective strategies to improve the uptake of cervical cancer screenings aong women in Lagos, Nigeria.

## Materials and methods

### Study design and settings

This was a prospective follow-up study conducted as part of the ‘mHealth-Cervix trial’ [16]. The study involved women who attended the general outpatient (GOP) clinics of Lagos University Teaching Hospital and Lagos State University Teaching Hospital, Nigeria, between August 2020 and April 2021. These are the two foremost healthcare institutions in Lagos that serve as referral centres for other government-owned and private hospitals in Lagos and its neighbouring states. The hospitals provide a variety of care including integrated gynaecologic oncology prevention services such as Pap smearing, human papillomavirus testing and colposcopy services, including biopsy and histology. The GOP clinics in the participating hospitals are opened each day of the week and attendees are mainly the National Health Insurance Scheme enrollees who have routine Pap smear screening as part of their standard health insurance coverage [18].

### Study population and eligibility criteria

The study participants were women in the control arm of the ‘mHealth-Cervix trial’ [16]. They were women aged 25–65 years without prior history of cervical premalignant or malignant lesions; those who have not had a Pap smear in the past 3 years; and those with sufficient physical and mental capacities to understand the purpose of the study. Women who were considering a change of residential relocation at any time in the following 6 months and those who failed to give consent at enrolment or withdrew their consent at follow-up were excluded from the study.

### Study procedures and data collection

Initial health education on cervical cancer and its prevention was given to all women attending the GOP clinics as part of the usual health promotion programme. Eligible women from the clinics were subsequently identified and invited to enrol in the ‘mHealth-Cervix trial’ [16]. After obtaining their baseline socio-demographic and clinical information, the women were then followed up for 6 months as indicated in the control arm of the trial [16]. The women’s monthly income and educational status were used as independent determinants of their socio-economic status according to the classification by Yoon *et al* [19]. Participants’ tracking was via phone calls and/or review of medical records in the 6 months follow-up period. The study endpoints were the predictive effects of socio-demographic and clinical factors on Pap smear screening uptake during 6 months of follow-up.

### Statistical methods

The sample size for the mHealth-Cervix trial [16] was calculated using G*Power for Windows version 3.1.9.2 (Kiel University, Germany). We extracted the data of the 100 women in the control arm of the trial to assess the influence of various socio-demographic and clinical factors on Pap smear testing uptake. Statistical analyses were carried out using SPSS version 27.0 for Windows (Armonk, NY: IBM Corp.). Descriptive statistics were presented as frequency (and percentage) and mean (± standard deviation) and the associations between categorical variables were tested using Pearson’s Chi-square (*X^2^*) test. Using the predictive model in a binary logistic regression analysis, we assessed the influence of various socio-demographic and clinical factors as predictors of Pap smear screening uptake. Statistical significance was reported as *p*-value < 0.05.

### Ethical considerations

The Health Research Ethics Committee of the College of Medicine, University of Lagos, approved the mHealth-Cervix trial with approval number: CMUL/HREC/12/19/704. We applied the ethical principles of Helsinki during the study. Following counselling, participants read and signed an informed consent form before their enrolment in the study. Strict confidentiality of participants’ information was ensured during and after the completion of the study.

## Results

Out of 100 women enrolled into the control arm of the mHealth-Cervix trial, 2 were lost to follow-up. As shown in Table 1, the mean age of the participants enrolled in the study was 38.0 ± 10.3 years and the mean residential location from the clinic was 15.1 ± 8.5 km. The participants were predominantly multiparous (*n* = 76, 77.6%), premenopausal (*n* = 77, 78.6%), married (*n* = 71, 72.4%), had at least secondary school education (*n* = 69, 70.4%) and of middle to upper income level (*n* = 59, 60.2%). A small proportion of the study participants were previously aware of Pap testing (*n* = 38, 38.8%), while an even smaller proportion had previously had Pap smear done (*n* = 27, 27.6%).

The Pap test uptake rate during the 6-month follow-up was 35.7% (*n* = 63) (Table 2). In the univariate binary logistic regression analysis, participants with middle- (*p* = 0.004) and upper-income (*p* = 0.003) levels, previous knowledge of Pap smear (*p* = 0.001), who had Pap smear testing in the past (*p* = 0.001) and those who attended the GOP clinics during the follow-up period for other various reasons (*p* = 0.001) were significantly more likely to have Pap smear testing. While keeping other covariates constant in the final multivariate model, participants’ previous awareness of Pap smearing (RR = 6.92, 95%CI: 8.37–56.68, *p* = 0.001) and GOP clinic attendance during their follow-up period (RR = 11.22, 95%CI: 1.54–81.51, *p* = 0.017) were the only independent predictors of Pap smear testing uptake in the study (Table 3).

## Discussion

The Pap test uptake rate recorded in this prospective follow-up study was 35.7%, and we found that participants’ prior knowledge of Pap smear as a means of cervical cancer screening and their GOP clinic attendance during the period of follow-up were significant independent predictors of Pap smear screening uptake.

The mean age of the participants in the study (38.0 ± 10.3 years) was only slightly higher than the mean ages of 35.7 ± 9.74 years [20] and 35.9 ± 9.5 [14] recorded in our previous studies that assessed the knowledge of cervical cancer prevention among women in Lagos. These findings suggest that clinic attendees who willingly participate in such clinical research in our settings are of a relatively younger age group.

We recorded a Pap smear uptake rate of 35.7% in this study and this is much higher than the previous testing rate of 22.9% reported in a similar clinic setting in Lagos [14] and much higher than the 3.2% rate recorded by Ajenifuja *et al* [12] in Ile-Ife. The higher screening uptake in this study overemphasised the importance of cervical cancer prevention education, an integral component of complementary health promotion, provided to potential participants at enrolment in clinics on the overall improvement in the women’s health-seeking behaviour.

Our study revealed no impact of the distance of the women’s homes to the participating health facilities and their uptake of Pap smear testing, and this corroborated the findings of several other studies that found no relationship between distance to nearest facility and healthcare utilisation [21]. This showed that in most contexts, other barriers to access, such as an individual’s perception of the disease, social support, time availability and health decision autonomy, may even be more important factors that affect health-seeking behaviour [22]. Similar to other previous studies conducted in Africa [14,16,23], the attainment of any form of Western education did not positively influence the uptake of Pap smear testing in this study. However, another Nigerian study by Wright et al [13] reported that having a formal education is a significant predictor of uptake of cervical cancer screening. These contrasting findings suggest that education, despite being a vital tool in healthcare promotion, may not necessarily be the sole determinant of an individual’s health-seeking behaviour. Surprisingly, in the current study, participants’ income status has no association with Pap test uptake and this is in contrast to the findings reported from the total participants’ data in the mHealth-Cervix trial that reported that women in the middle- and upper-income statuses are significantly more likely to have Pap smear testing done [16]. This is because women of higher income levels are more financially empowered to access healthcare information and take healthcare decisions, leading to healthcare service uptake independent of any other factors.

Women with previous awareness of Pap testing showed an increased Pap smear uptake in the current study and this corroborated the findings from our previous study [16], which thus suggests that the provision of health education on cervical cancer prevention by healthcare professionals at any time in the regular clinics will reinforce previous knowledge of Pap smear screening which will further increase the likelihood of uptake of Pap testing among women. As GOP clinic attendance for various other reasons during the period of follow-up significantly influenced the Pap smear screening uptake among women in the study, it further suggests that the constraint for time usually pushes many individuals to use the opportunity of visiting the healthcare facilities during infirmities to seek other necessary and accessible healthcare prevention services [16].

The study is mainly limited in terms of generalisation as it was conducted within the hospital settings. In addition, the 6-month period adopted for follow-up in the study may be too short to assess the impact of long-term behavioural changes following the pre-enrolment health education on the real-world uptake of cervical cancer screening. Nevertheless, the data provide useful data on the potential factors that may predict women’s uptake of Pap smear screening after a routine and targeted cancer prevention education by healthcare providers in healthcare facilities in low- and middle-income settings.

## Conclusion

In the cohort of participants from this current study, we reported that a woman’s prior knowledge of Pap smear testing and her attendance at the GOP clinic for other healthcare consultations had a positive influence on her Pap smear screening uptake. We will, therefore, aim to further explore the long-term impact of continuous health education and promotion on cervical cancer prevention and its effect in the context of routine clinical care. This will be introduced alongside methods to increase the flow of information and value of content for women in the real world and hospital settings. In the meantime, we recommend that regular health education on cervical cancer prevention be further reinforced as an integral part of complementary health promotion by healthcare providers in the clinics to reduce the overall high burden of cervical cancer incidence and deaths in most low- and middle-income settings.

## Conflicts of interest

The authors have no conflicts of interest.

## Funding declaration

This work was partly supported by the Fogarty International Centre of National Institutes of Health (Award Numbers D43TW010934, D43TW010134 and D43TW010543) and the Conquer Cancer International Innovation Grant (Project ID 16576). The views expressed by the authors are theirs alone and do not reflect the official views of the National Institutes of Health and the American Society of Clinical Oncology® or Conquer Cancer®.

## Figures and Tables

**Table 1.** Baseline characteristics of participants at enrolment (*n* = 98).^a^

Characteristic	*n*(%)
Age (years)	38.0 ± 10.3
Residential location from the clinic (km)	15.1 ± 8.5
**Prior pregnancies**	
0	15 (15.3)
1-3	76 (77.6)
>3	7 (7.1)
**Menopausal status**	
Premenopause	77 (78.6)
Postmenopause	21 (21.4)
**Marital status**	
Never married	18 (18.4)
Married	71 (72.4)
Divorced	6 (6.1)
Widowed	3 (3.1)
**Level of education**	
No formal education	7 (7.1)
Primary education	22 (22.4)
Secondary education	41 (41.8)
Tertiary education	28 (28.6)
**Level of income**	
Lower	39 (39.8)
Middle	36 (36.7)
Upper	23 (23.5)
**Awareness of Pap smear**	
Yes	38 (38.8)
No	60 (61.2)
**Previous Pap smear testing uptake**	
Yes	27 (27.6)
No	71 (72.4)

km, kilometre; Pap, Papanicolaou

a Values are given as mean ± SD or number (percentage) unless indicated otherwise

**Table 2.** Univariate analyses of socio-demographic and clinical predictors of Pap smear testing uptake after 6 months of follow-up (*n* = 98)

Predictors	Estimates of effect on Pap smear testing uptake			
	Uptake *n* = 35 (%)	Non-uptake *n* = 63 (%)	Risk ratio (95%CI)	*p*-value
**Age, years**				
**≥38**	14 (35.0)	26 (65.0)	1.00 (ref)	-
**<38**	21 (36.2)	37 (63.8)	1.05 (0.45–2.46)	0.902
**Residential location, km**				
**≥15**	17 (32.7)	35 (67.3)	1.00 (ref)	-
**<15**	18 (39.1)	28 (60.9)	1.32 (0.58–3.03)	0.507
**Prior pregnancies**				
**0**	8 (53.3)	7 (46.7)	1.00 (ref)	-
**1**–**3**	24 (31.6)	52 (68.4)	1.52 (0.25–9.29)	0.648
**>3**	3 (42.9)	4 (57.1)	0.62 (0.60–2.97)	0.545
**Menopausal status**				
**Premenopause**	28 (36.4)	49 (63.6)	1.00 (ref)	-
**Postmenopause**	7 (33.3)	14 (66.7)	1.09 (0.56–2.14)	0.797
**Marital status**				
**Never married**	7 (38.9)	11 (61.1)	1.00 (ref)	-
**Ever married**	28 (35.0)	52 (65.0)	0.85 (0.29–2.43)	0.756
**Educational status**				
**No formal education**	2 (28.6)	5 (71.4)	1.00 (ref)	-
**Educated**	33 (36.3)	58 (63.7)	1.42 (0.26–7.74)	0.684
**Level of income**				
**Lower**	6 (15.4)	33 (84.6)	1.00 (ref)	-
**Middle**	17 (47.2)	19 (52.8)	4.92 (1.66–14.61)	0.004
**Upper**	12 (52.2)	11 (47.8)	6.00 (1.81–19.81)	0.003
**Awareness of Pap smear**				
**No**	3 (5.0)	57 (95.0)	1.00 (ref)	-
**Yes**	32 (84.2)	6 (15.8)	16.84 (5.54–51.18)	0.001
**Previous Pap smear testing**				
**No**	11 (15.5)	60 (84.5)	1.00 (ref)	-
**Yes**	24 (88.9)	3 (11.1)	5.74 (3.28–10.04)	0.001
**GOP clinic attendance**				
**No**	16 (21.6)	58 (78.4)	1.00 (ref)	-
**Yes**	19 (79.2)	5 (20.8)	13.78 (4.45–42.64)	0.001

**km, kilometre; GOP, general outpatient; Pap, Papanicolaou**

**Table 3.** Multivariate analyses of predictors of Pap test uptake after 6 months of follow-up (*n* = 98)

Predictors	Estimates of effect on Pap smear testing uptake	
	Risk ratio (95%CI)	*p*-value
**Level of income**		
**Lower**	1.00 (ref)	-
**Middle**	2.96 (0.36–24.40)	0.313
**Upper**	2.97 (0.50–17.59)	0.230
**Awareness of Pap smear**		
**No**	1.00 (ref)	0.001
**Yes**	6.92 (8.37–56.68)	-
**Previous Pap smear testing**		
**No**	1.00 (ref)	-
**Yes**	1.43 (0.18–11.30)	0.737
**GOP clinic attendance**		
**No**	1.00 (ref)	-
**Yes**	11.22 (1.54–81.51)	0.017

**GOP, general outpatient; Pap, Papanicolaou**
